# Pseudoxanthoma elasticum: Die Lotsenfunktion des Dermatologen

**DOI:** 10.1007/s00105-022-04987-6

**Published:** 2022-04-15

**Authors:** Jana Burghaus, Roland Schneiderbauer, Ferdinand Toberer

**Affiliations:** 1 Abteilung für Dermatologie, Venerologie und Allergologie, Universitätsklinikum Heidelberg, Im Neuenheimer Feld 440, 69120 Heidelberg, Deutschland; 2 Haut- und Laserzentrum Rosenheim, 83022 Rosenheim, Deutschland

**Keywords:** ABCC6, Hereditäre ektope Mineralisationsstörung, Metabolische Erkrankung, Hydroxylapatit-Kristalle, Pyrophosphat, ABCC6, Heritable ectopic mineralization disorder, Metabolic disease, Calcium hydroxyapatite crystals, Pyrophosphate

## Abstract

Charakteristische Hautveränderungen führen zu der Diagnose eines Pseudoxanthoma elasticum (PXE). Das PXE repräsentiert eine ektope Mineralisationsstörung, welche primär die Haut, die Augen und das arterielle Gefäßsystem betrifft. Eine frühzeitige Diagnosestellung ist entscheidend für die rechtzeitige Behandlung von extrakutanen Komplikationen. Wir verdeutlichen die Lotsenfunktion der Dermatologen und Dermatologinnen anhand einer Serie von vier unabhängigen Fällen eines PXE mit pathognomonischen Hautveränderungen und einem weiten Spektrum an systemischen Komplikationen.

## Abstract (en)

Characteristic skin changes lead to diagnosis of pseudoxanthoma elasticum (PXE), an ectopic mineralization disorder, involving primarily the skin, eyesight, and arterial vessels. Early recognition is crucial for timely treatment of extracutaneous complications. We hereby report a series of four cases of PXE with pathognomonic skin lesions and a broad spectrum of systemic complications.

## Falldarstellungen

### Anamnese und klinische Präsentation

#### Patient 1

**Figure 1:**
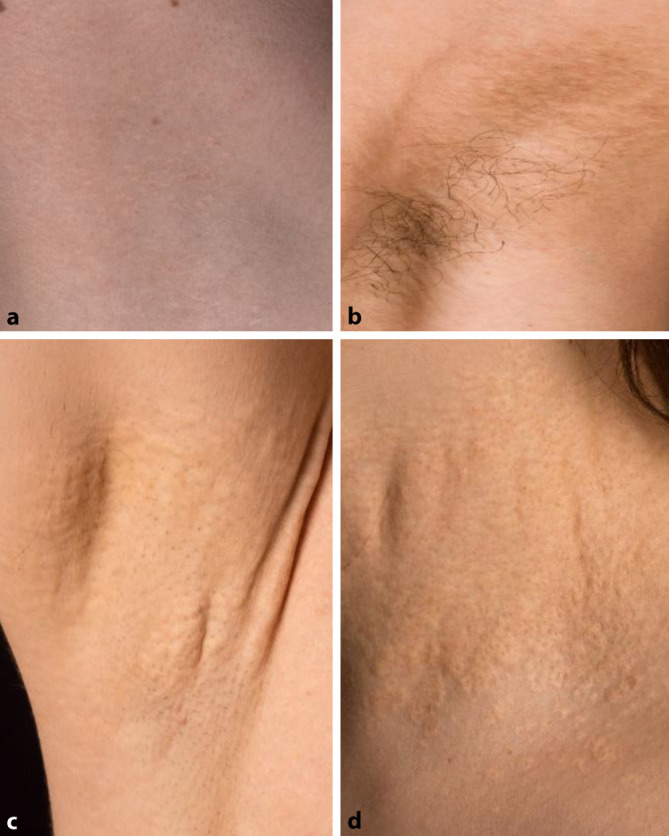

Ein 14-jähriger Junge stellte sich mit asymptomatischen, gelben Papeln am seitlichen Hals vor, welche vor einem Jahr erstmals aufgefallen waren (Abb. 1a). Die Großmutter des Patienten wies ähnliche Papeln auf.

#### Patientin 2

Eine 21 Jahre alte Frau wurde in unserer dermatologischen Ambulanz mit gelblichen Papeln am Hals und an beiden Achseln vorstellig, welche bereits seit 7 Jahren eine langsame Progredienz zeigten (Abb. 1b). Der Bruder der Patientin war ebenfalls betroffen.

#### Patient 3

Ein 60-jähriger Mann, welcher in der Vorgeschichte bereits einen Apoplex erlitten hatte, fiel in der dermatologischen Untersuchung mit einem irregulären Oberflächenrelief am lateralen Hals auf.

#### Patientin 4

Eine 35 Jahre alte Frau bemerkte eine vermehrte Schlaffheit der Haut und zunehmende, gelbliche Papeln, welche sich zervikal, axillär und periumbilikal zeigten (Abb. 1c, d).

## Befund

Die kardiologische und ophthalmologische Untersuchung der Patienten 1, 2 und 4 waren bei der Erstvorstellung unauffällig. Der Patient 3 wies ein kardiovaskuläres Risikoprofil mit Zustand nach Apoplex und koronarer Herzerkrankung auf, eine Claudicatio wurde jedoch verneint, und er präsentierte ebenfalls keine ophthalmologischen Auffälligkeiten.

**Figure 2:**
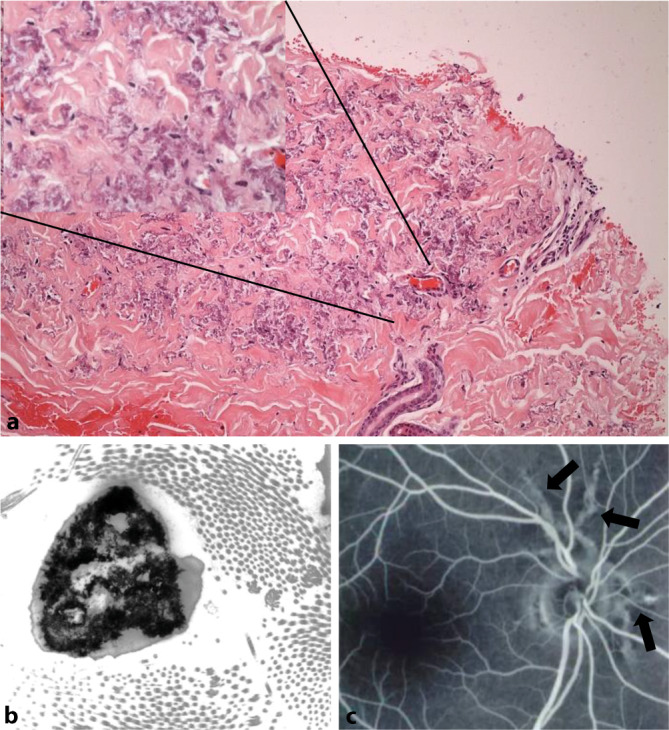

Zur weiteren diagnostischen Einschätzung erfolgte eine histologische Untersuchung einer Hautbiopsie der zervikalen Papeln von Patient 2. Es zeigten sich basophile plumpe, elastische Fasern in der retikulären Dermis (Abb. 2a). Einen gleichartigen Befund hatte zuvor auch eine bereits extern erfolgte Histologie der Papeln am Hals von Patient 1 ergeben. Eine zervikale Hautstanze von Patient 3 hingegen wurde elektronenmikroskopisch untersucht und erbrachte elektronendichte Mineralablagerungen in der Elastinkomponente der retikulären Dermis (Abb. 2b).

Patient 4 erhielt keine Histologie, aber eine humangenetische Untersuchung, in welcher eine Mutation im *ABCC6*-Gen festgestellt werden konnte.

## Diagnose

In allen 4 dargestellten Fällen konnte somit in Einklang mit Histologie, Elektronenmikroskopie und Humangenetik die Diagnose eines Pseudoxanthoma elasticum (PXE) gestellt werden.

Im Falle des Patienten 3 wurde bei Zustand nach Apoplex der Verdacht auf ein PXE mit Beteiligung der arteriellen Gefäße geäußert.

## Therapie und Verlauf

Alle vier Patienten erhielten ab der Diagnosestellung engmaschige kardiologische und ophthalmologische Vorsorgeuntersuchungen. In diesem Zusammenhang wurden drei Jahre später in der Fundoskopie von Patientin 4 sog. „angioid streaks“ sichtbar (Abb. 2c).

Eine Therapie der dermatologischen Veränderungen erfolgte in keinem der gezeigten Fälle.

## Diskussion

Das PXE stellt eine autosomal-rezessiv vererbte, ektope Mineralisationsstörung dar, welche durch Mutationen des „ATP binding cassette subfamily C member 6“(*ABCC6*)-Gens ausgelöst wird. Das *ABCC6*-Gen kodiert für eine Effluxpumpe, welche primär in der Leber vorhanden ist. Das PXE wird charakterisiert durch eine Kalzifikation der elastischen Fasern in verschiedensten Organen, symptomatisch werden diese jedoch nur in der Haut, arteriellen Gefäßen und der okulären Bruch-Membran [2, 5]. Die Prävalenz in der Allgemeinbevölkerung liegt zwischen 1:25.000 und 1:56.000 [8].

Durch den gestörten Efflux eines bislang unbekannten Substrates wird die ATP-Ausscheidung in der Leber vermindert [3], was zu einer reduzierten Menge an Pyrophosphat (PPi), einem starken Inhibitor der ektopen Mineralisation [5, 8], führt.

Dermatologisch sind überwiegend der Hals, die Achseln, Armbeugen und der Umbilicus von einer vermehrten Schlaffheit der Haut mit verminderter Flexibilität und Auftreten gelblicher oder cremefarbener Papeln betroffen. Die charakteristischen Hautveränderungen manifestieren sich jedoch trotz der genetischen Grundlage häufig erst im zweiten bis dritten Lebensjahrzehnt und können zudem auch an weniger offensichtlichen Stellen wie der Mundschleimhaut oder seltener auch der Genitalschleimhaut imponieren [2].

Zur Sicherung der klinischen Verdachtsdiagnose genügt der histologische Nachweis der pathognomonischen Kalzifizierung der fragmentierten elastischen Fasern in der mittleren Dermis in der Von-Kossa-Färbung.

Bezüglich der dermatologischen Therapie finden sich in der Literatur nur wenige Berichte über kosmetische Operationen, Kollageninjektionen und Skin-Resurfacing mit einem fraktionierten CO_2_-Laser. Insgesamt waren die erzielten Verbesserungen mit einer hohen Rezidivrate oder Komplikationen assoziiert, sodass die Therapieindikation zurückhaltend gestellt werden sollte [8].

Frühzeitig behandlungsbedürftig hingegen sind die ophthalmologischen und kardiovaskulären Komplikationen des PXE, da sie weitreichende, teils auch letale Konsequenzen für die betroffenen Patienten haben können. Eine zunehmende Kalzifikation führt zu Rissen in der Bruch-Membran, welche als „angioid streaks“ fundoskopisch sichtbar werden. Neovaskularisationen sind die Folge, und ein Verlust des Augenlichts droht. Der Visusverlust kann jedoch effektiv und komplikationsarm verhindert werden durch eine frühzeitige intravitreale Applikation von „vascular endothelial growth factor“(VEGF)-Inhibitoren, welche die Gefäßneubildung hemmen [6].

Die arterielle Kalzifikation betrifft v. a. die unteren Extremitäten und führt zu einer symptomatischen Claudicatio. Außerdem ist das Risiko für einen Myokardinfarkt oder Apoplex erhöht. Die prophylaktische Behandlung der PXE-Patienten konzentriert sich auf eine Senkung der Lipidwerte im Serum, eine regelmäßige Bewegungstherapie und insgesamt eine Reduktion weiterer kardiovaskulärer Risikofaktoren.

Es gibt keine gesamtheitliche Therapie des PXE. Eine hoch dosierte Einnahme von Magnesium kann einen leicht positiven Effekt haben [4, 7]. Des Weiteren zeigten Bisphosphonate einen unterstützenden Effekt und konnten vaskuläre Kalzifikationen außerhalb der Koronararterien reduzieren [8].

Keiner der vielfältigen weiteren therapeutischen Versuche (z. B. reduzierte Kalziumaufnahme, Phosphatbinder, Vitamin-E-, -C- oder -K-Supplementation, Natriumthiosulfat) zeigte einen bedeutenden Einfluss auf die kardiovaskulären oder okulären Symptome [8].

Aktuell fokussiert sich die Forschung vermehrt auf eine molekulargenetische Therapie des PXE. So konnte bereits in einem Mausmodel das *ABCC6*-Gen erfolgreich mithilfe eines adenoviralen Vektors übertragen werden und führte zu einer verminderten ektopen Kalzifikation [1]. Als weiterer Therapieversuch wurde das Chaperon Natriumphenylbutyrat (4-PBA) im Mausmodel eingesetzt, um fehlgefalteten *ABCC6*-Mutationen mit erhaltener Transportaktivität zur erfolgreichen Lokalisierung im Bereich der Plasmamembran zu verhelfen. Zudem wurde 1,2,4-Oxadiazol (PTC-124) als Substanz zur Unterdrückung eines vorzeitigen Stopcodons im Rahmen der Translation getestet [8, 9]. Weitere Studien sind jedoch nötig, bevor eine molekulargenetische Therapie des PXE für Menschen zur Verfügung stehen kann.

Zusammengefasst soll diese Fallserie mit der Klinik und Diagnostik des PXE vertraut machen und betonen, dass dem Dermatologen/der Dermatologin im Falle des PXE eine zentrale Lotsenfunktion zufällt. Eine zeitnahe Diagnosestellung aufgrund der charakteristischen Hautveränderungen ermöglicht somit eine frühzeitige interdisziplinäre Patientenführung, wodurch schwerwiegende extrakutane Komplikationen des PXE rechtzeitig behandelt werden können.

## Fazit für die Praxis

- Das Pseudoxanthoma elasticum stellt eine hereditäre, ektope Mineralisationsstörung dar.
- Unbehandelt drohen ein Visusverlust und kardiovaskuläre Komplikationen (Myokardinfarkt, Apoplex, Hypertonie bei Befall der Nierenarterien).
- Dem Dermatologen/der Dermatologin kommt eine entscheidende Lotsenfunktion zu, da eine frühe Diagnosestellung über gelbliche Papeln an Hals und Achseln eine rechtzeitige Therapie der systemischen Komplikationen ermöglicht.
- Intravitreal werden VEGF-Inhibitoren appliziert.
- Kardiovaskulär wird eine Lipidsenkung angestrebt.
